# Enhancing Antidiabetic Drug Selection Using Transformers: Machine-Learning Model Development

**DOI:** 10.2196/67748

**Published:** 2025-06-02

**Authors:** Hisashi Kurasawa, Kayo Waki, Tomohisa Seki, Eri Nakahara, Akinori Fujino, Nagisa Shiomi, Hiroshi Nakashima, Kazuhiko Ohe

**Affiliations:** 1 The University of Tokyo Hospital, 7-3-1 Hongo, Bunkyo-ku, Tokyo, Japan, 81 3-5800-6427; 2 Nippon Telegraph and Telephone Corporation, Japan

**Keywords:** AI, diabetes, drug selection, machine learning, transformer, artificial intelligence

## Abstract

**Background:**

Diabetes affects millions worldwide. Primary care physicians provide a significant portion of care, and they often struggle with selecting appropriate medications.

**Objective:**

This study aimed to develop a model that accurately predicts what drug an endocrinologist would prescribe based on the current measurements. The goal was to create a system that would assist nonspecialists in choosing medications, thereby potentially improving diabetes treatment outcomes. Based on the performance of previous studies, we set a performance target of achieving a receiver operating characteristic area under the curve (ROC-AUC) above 0.95.

**Methods:**

A transformer-based encoder-decoder model predicts whether 44 types of diabetes drugs will be prescribed. The model uses sequences of age, sex, history for 12 laboratory tests, and prescribed drug history as inputs. We assessed the model using the electronic health records from 7034 patients with diabetes seeing endocrinologists between 2012 and 2022 at the University of Tokyo Hospital. We assessed model performance trained on data subsets spanning different time periods (2, 5, and 10 years) using micro- and macro-averaged ROC-AUC on a hold-out test set comprising data solely from 2022. The model’s performance was compared against LightGBM.

**Results:**

The model trained on data from the past 5 years (2017‐2021) yielded the best predictive performance, achieving a microaverage (95% CI) ROC-AUC of 0.993 (0.992-0.994) and a macroaverage (95% CI) ROC-AUC of 0.988 (0.980-0.993). The model achieved an ROC-AUC above 0.95 for 43 out of 44 drugs. These results surpassed the predefined performance target and outperformed both previous studies and the LightGBM model’s microaverage ROC-AUC of 0.988 (0.985-0.990) in terms of prediction accuracy. Furthermore, training the model with short-term data from the past 5 years yielded high accuracy compared to using data from the past 10 years, suggesting that learning from more recent prescribing patterns might be advantageous.

**Conclusions:**

The proposed model demonstrates the feasibility of accurately predicting the next prescribed drugs. This model, trained from the past prescriptions of endocrinologists, has the potential to provide information that can assist nonspecialists in making diabetes-treatment decisions. Future studies will focus on incorporating important factors such as prescription contraindications and constraints to enhance safety, as well as leveraging large-scale clinical data across multiple hospitals to improve the generalizability of the model.

## Introduction

Diabetes affects 529 million people worldwide, with 1 in every 10 adults experiencing the condition [1]. Patients with diabetes receive care from primary care physicians, not endocrinologists, in many areas including the United States, Europe [2], and Japan [3]. This is particularly concerning in the United States, where the endocrinologist shortage is significant: the population-to-endocrinologist ratio within 20 miles was 29,887:1 for adults aged 18‐64. Rural areas face even greater disparities, with only 55.5% of adults having access to at least 1 endocrinologist within that distance [4]. Japan also faces a similar problem. Japan has 11 million patients with diabetes but only about 7000 specialists, and two-thirds of people with type 2 diabetes (T2D) receive care from primary care physicians [3]. These nonspecialists may struggle to predict a patient’s glycemic control. Approximately 60% of surveyed patients with T2D treated by nonspecialists experienced poor glycemic control (hemoglobin A_1c_ [HbA_1c_] ≥8%), with around 30% seeing worsened levels the following year, according to a survey on T2D treatment practices by primary care physicians [3].

One of the difficulties in diabetes treatment for primary care physicians is drug selection [5,6]. Medications are prescribed either as monotherapy or in combination. Medications need to be chosen [7] considering insulin secretion and insulin resistance [8], age [9,10], degree of obesity [11], severity of chronic complications [12], liver function, and kidney function [13]. New diabetes treatments continue to be developed, expanding the options for medication selection. For example, in Japan, sodium–glucose cotransporter 2 [14], imeglimin [15], and tirzepatide [16] were introduced in 2014, 2021, and 2023, respectively. The selection of diabetes medications depends on individual patient factors, with guidelines [17] and treatment tendencies [18] that vary from country to country. A tool supporting drug selection could enhance treatment outcomes. It could provide early warning to physicians and potentially improve treatment outcomes for patients who are being examined by nonspecialized physicians.

Clinical decision support systems (CDSS) provide physicians with safety tools for drug selection [19]. By adopting a knowledge-based approach aligned with clinical guidelines, CDSS help to prevent medication errors such as overdosing, incomplete, or unclear orders [20,21]. However, replicating the ability of endocrinologists to select drugs for individual patient conditions in a CDSS is complex and challenging for knowledge-based approaches.

Machine learning (ML) has demonstrated success in predicting patient symptoms, including forecasting the onset of T2D [22] and predicting complications [23]. Several studies have applied ML to the problem of drug selection for patients with diabetes [24]. A study predicting the next prescribed diabetes drugs for 161,497 patients with diabetes using sequential pattern mining demonstrated an accuracy of 89.1%‐90.5% in guessing from 37 drug classes (eg, DPP-4 inhibitor) and 63.5%‐64.9% accuracy in guessing from 43 drugs (eg, algliptin) [25]. Another study predicted 7 drug classes with an ROC-AUC of 90.6%‐94.3% using recurrent neural networks (RNN) [26]. There are also proposed methods for predicting treatment outcomes after selecting diabetes medications [27].

The field of ML has undergone significant advancement since the introduction of the transformer approach in 2017 [28,29]. Transformers enable contextual interactions for natural language processing tasks and have become a core technology across diverse domains [30-32]. The transformer model incorporates an attention mechanism and has shown remarkable performance in tasks involving the extraction of temporal and semantic relationships, leading to success in tasks such as generation and classification [33]. Generally, transformers enhance predictive performance through two types of training: pretraining via self-supervised learning and fine-tuning via supervised learning. For example, large language models like GPT and bidirectional encoder representations from transformers (BERT) were pretrained on a task predicting the next or masked word and then fine-tuned on a task generating responses to an instruction [34]. Several studies in health care have already used this scheme of 2 types of learning. TransformEHR improved performance on the fine-tuned task of predicting the onset of pancreatic cancer and intentional self-harm among patients with posttraumatic stress disorder by pretraining on the task of predicting randomly-masked diseases and outcomes in time series of 6.5 million patients [35]. A deep neural sequence transduction model for electronic health records (BEHRT) [36] was pretrained using an electronic health records (EHR) dataset of 1.6 million patients and was fine-tuned to predict diagnosis codes. Recent transformers have also made advancements in learning by combining different modalities of information such as images, audio, and text. Foresight [37] was trained with structured data such as laboratory results as well as unstructured data such as free text from 1.5 million patients across three EHR datasets. The transformer approach has not previously been applied to the task of selecting diabetes medications. In addition, while efforts have been made to train models with large amounts of data to improve accuracy [38], the impact of the training data period on predictions is largely unexplored.

**Figure 1.**
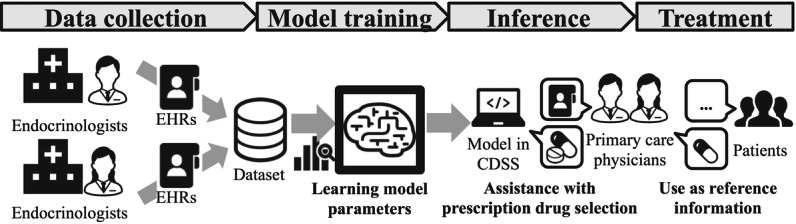
System image of prescription drug selection assistant.

Diabetes drug selection involves deciding to prescribe one or more drugs from among many candidates. This selection can be handled by ML as a multichoice task. This study aimed to develop an ML tool that accurately predicts the next prescribed drugs using the patient’s medical condition and prescription history over the past year. The objective is to enhance diabetes treatment outcomes for nonspecialists through improved support in drug selection (Figure 1). Based on the performance of previous studies [24-26], our goal was to achieve an ROC-AUC above 0.95 when predicting the next prescribed drugs. Drawing on our team’s previous work in self-management support for T2D treatment [39] and predicting treatment discontinuations [40,41], we designed this task with the hope of overcoming barriers to nonspecialist diabetes treatment in clinical practice, believing it could significantly improve diabetes treatment outcomes.

## Methods

### Datasets

All data were collected from the EHRs at the University of Tokyo Hospital, which included 7034 patients who visited the hospital, had diagnostic codes, and were registered to the Japan diabetes comprehensive database project based on an advanced electronic medical record system (J-DREAMS) cohort [42]. The data were recorded in the EHRs between January 1, 2011 and December 31, 2022. The data, including treatment decisions and outcomes, were reflective of care by endocrinologists. Variables extracted from the EHRs included sex, age, 12 kinds of diabetes-related laboratory tests, and drugs. The laboratory tests included numerical values of HbA_1c_, glucose, triacylglycerol, high density lipoprotein cholesterol, total cholesterol [43], urinary albumin creatinine ratio, creatinine [44], alanine transaminase, aspartate transaminase, and γ-glutamyltransferase [45], and categorical values of proteins [45] and glycogen. Drugs were identified using the list of drug price standards [46] provided by the Ministry of Health, Labour and Welfare of Japan, and 44 types prescribed in 2021 were selected.

### Training and Test Data

The records used for training were not used for testing to ensure that the same patients were not included in both groups. A total of 80% (5627/7034) of patients were included in the training group, and the remaining 20% (1407/7034) of patients were included in the testing group.

In order to examine the size of the training data needed to achieve the target prediction accuracy, we extracted three different subsets of training data from the sequences of the 5627 patients in the training group: 2 years from 2020 to 2021 (3013 patients and their 25484 subsequences), 5 years from 2017 to 2021 (4009 patients and their 78,020 subsequences), and 10 years from 2012 to 2021 (4524 patients and their 168,595 subsequences). For testing, we further identified a subset of the testing data that was solely data from 2022 (637 patients and their 2988 subsequences). Thus, there was no overlap in patients or time periods between training and testing. Table 1 shows the characteristics of the patients. The drugs in the table are the top 5 and bottom 5 in terms of number of prescriptions in the 2-year training data. All characteristics are provided in Multimedia Appendix 1.

**Table 1.** Characteristics of patients with diabetes included in the training and testing datasets. Characteristics are presented separately for the patient groups belonging to the different training datasets defined by period: 2 years (2020-2021), 5 years (2017-2021), 10 years (2012-2021) and the independent test dataset (data exclusively from 2022).

	2 years of training data		5 years of training data		10 years of training data		1 year of test data	
	Records (n=25484)	Patient (n=3013)	Records (n=78020)	Patient (n=4009)	Records (n=168595)	Patient (n=4524)	Records (n=2988)	Patient (n=637)
Sex								
Male, n (%)	16224 (63.66)	1915 (63.56)	49348 (63.25)	2543 (63.43)	106782 (63.34)	2869 (63.42)	1793 (60.01)	381 (59.81)
Female, n (%)	9260 (36.34)	1098 (36.44)	28672 (36.75)	1466 (36.57)	61813 (36.66)	1655 (36.58)	1195 (39.99)	256 (40.19)
Age, mean (SD)	67.62 (12.59)	69.06 (12.50)	67.25 (12.56)	69.17 (12.87)	66.41 (12.30)	68.98 (13.10)	68.80 (12.31)	69.68 (12.26)
HbA1c^a^								
Mean (SD)	7.37 (1.08)	—^b^	7.31 (1.06)	—	7.25 (1.03)	—	7.31 (1.02)	—
<6, n (%)	920 (3.61)	323 (10.72)	3046 (3.90)	754 (18.81)	7286 (4.32)	1345 (29.73)	96 (3.21)	49 (7.69)
6‐7, n (%)	8874 (34.82)	1865 (61.90)	29030 (37.21)	3027 (75.51)	66470 (39.43)	3835 (84.77)	1113 (37.25)	370 (58.08)
7‐8, n (%)	10177 (39.93)	2135 (70.86)	30493 (39.08)	3157 (78.75)	64355 (38.17)	3830 (84.66)	1189 (39.79)	404 (63.42)
≥8, n (%)	5513 (21.63)	1224 (40.62)	15451 (19.80)	2008 (50.09)	30484 (18.08)	2725 (60.23)	590 (19.75)	200 (31.40)
missing, n (%)	0 (0.00)	0 (0.00)	0 (0.00)	0 (0.00)	0 (0.00)	0 (0.00)	0 (0.00)	0 (0.00)
HDL-C^c^								
Mean (SD)	60.99 (18.23)	—	60.07 (17.94)	—	59.81 (17.80)	—	63.76 (19.79)	—
<40, n (%)	1943 (7.62)	512 (16.99)	6633 (8.50)	964 (24.05)	14694 (8.72)	1374 (30.37)	158 (5.29)	67 (10.52)
40‐120, n (%)	21304 (83.60)	2737 (90.84)	63249 (81.07)	3644 (90.90)	134924 (80.03)	4195 (92.73)	2564 (85.81)	577 (90.58)
≥120, n (%)	195 (0.77)	59 (1.96)	494 (0.63)	100 (2.49)	966 (0.57)	137 (3.03)	44 (1.47)	11 (1.73)
missing, n (%)	2042 (8.01)	178 (5.91)	7644 (9.80)	243 (6.06)	18011 (10.68)	219 (4.84)	222 (7.43)	40 (6.28)
Cre^d^								
Mean (SD)	0.97 (0.69)	—	0.96 (0.75)	—	0.95 (0.72)	—	1.04 (1.08)	—
Male								
Mean (SD)	1.08 (0.71)	—	1.07 (0.74)	—	1.05 (0.74)	—	1.18 (1.23)	—
<0.65, n (%)	758 (2.97)	191 (6.34)	2675 (3.43)	405 (10.10)	5783 (3.43)	566 (12.51)	111 (3.71)	37 (5.81)
0.65‐1.09, n (%)	10672 (41.88)	1482 (49.19)	32766 (42.00)	2057 (51.31)	71870 (42.63)	2456 (54.29)	1145 (38.32)	290 (45.53)
≥1.09, n (%)	4543 (17.83)	750 (24.89)	13140 (16.84)	1093 (27.26)	27001 (16.02)	1337 (29.55)	516 (17.27)	129 (20.25)
missing, n (%)	251 (0.98)	6 (0.20)	767 (0.98)	5 (0.12)	2128 (1.26)	7 (0.15)	21 (0.70)	1 (0.16)
Female								
Mean (SD)	0.78 (0.60)	—	0.78 (0.72)	—	0.77 (0.65)	—	0.82 (0.74)	—
<0.46, n (%)	357 (1.40)	89 (2.95)	1160 (1.49)	175 (4.37)	2616 (1.55)	282 (6.23)	28 (0.94)	11 (1.73)
0.46‐0.82, n (%)	6596 (25.88)	895 (29.70)	20561 (26.35)	1224 (30.53)	44130 (26.18)	1428 (31.56)	825 (27.61)	194 (30.46)
≥0.82, n (%)	2133 (8.37)	372 (12.35)	6370 (8.16)	596 (14.87)	13572 (8.05)	730 (16.14)	326 (10.91)	93 (14.60)
missing, n (%)	174 (0.68)	11 (0.37)	581 (0.74)	11 (0.27)	1495 (0.89)	16 (0.35)	16 (0.54)	2 (0.31)
Glu^e^								
Mean (SD)	147.68 (51.19)	—	147.00 (51.31)	—	144.12 (50.77)	—	145.47 (48.67)	—
<70, n (%)	302 (1.19)	169 (5.61)	979 (1.25)	403 (10.05)	2709 (1.61)	743 (16.42)	28 (0.94)	22 (3.45)
70‐110, n (%)	4230 (16.60)	1389 (46.10)	13604 (17.44)	2483 (61.94)	33633 (19.95)	3403 (75.22)	555 (18.57)	260 (40.82)
≥110, n (%)	20863 (81.87)	2924 (97.05)	63201 (81.01)	3907 (97.46)	131586 (78.05)	4418 (97.66)	2394 (80.12)	609 (95.60)
missing, n (%)	89 (0.35)	7 (0.23)	236 (0.30)	5 (0.12)	667 (0.40)	6 (0.13)	11 (0.37)	2 (0.31)
Prescribed drug (top 5)								
Metformin hydrochloride, n (%)	11257 (44.17)	1390 (46.13)	33442 (42.86)	1952 (48.69)	72337 (42.91)	2376 (52.52)	1361 (45.55)	297 (46.62)
Sitagliptin phosphate hydrate, n (%)	4963 (19.47)	693 (23.00)	15420 (19.76)	1148 (28.64)	37438 (22.21)	1808 (39.96)	425 (14.22)	101 (15.86)
Insulin aspart (genetical recombination), n (%)	3212 (12.60)	377 (12.51)	10039 (12.87)	603 (15.04)	20149 (11.95)	833 (18.41)	403 (13.49)	85 (13.34)
Glimepiride, n (%)	2885 (11.32)	398 (13.21)	10387 (13.31)	690 (17.21)	28855 (17.11)	1141 (25.22)	358 (11.98)	76 (11.93)
Pioglitazone hydrochloride, n (%)	2731 (10.72)	354 (11.75)	9450 (12.11)	592 (14.77)	24305 (14.42)	910 (20.11)	244 (8.17)	53 (8.32)
Prescribed drug (buttom 5)								
Saxagliptin hydrate, n (%)	158 (0.62)	17 (0.56)	586 (0.75)	41 (1.02)	960 (0.57)	52 (1.15)	30 (1.00)	6 (0.94)
Anagliptin, n (%)	142 (0.56)	17 (0.56)	528 (0.68)	34 (0.85)	986 (0.58)	51 (1.13)	5 (0.17)	1 (0.16)
Insulin lispro (genetical recombination) [Insulin lispro Biosimilar 1], n (%)	59 (0.23)	19 (0.63)	59 (0.08)	19 (0.47)	59 (0.03)	19 (0.42)	58 (1.94)	13 (2.04)
Insulin glargine (genetical recombination) [Insulin glargin biosimilar 2], n (%)	39 (0.15)	9 (0.30)	86 (0.11)	14 (0.35)	86 (0.05)	14 (0.31)	1 (0.03)	1 (0.16)
Glibenclamide, n (%)	33 (0.13)	5 (0.17)	280 (0.36)	24 (0.60)	1722 (1.02)	87 (1.92)	11 (0.37)	3 (0.47)

a HbA_1c_: hemoglobin A_1c_.

b not applicable.

c HDL-C: high density lipoprotein cholesterol.

d Cre: creatinine..

e Glu: glucose.

### ML Models

Patients’ medical conditions and prescription histories are in general irregularly spaced, reflecting variability in patient care appointment dates, with updates to outpatient EHR occurring before and after clinical visits. We organized the data into Monday-to-Sunday weeks and quantized the data to a single value per week, using the average in the case of multiple measurements and treating weeks with no values as having missing values [47]. This approach allowed the ML model to treat irregularly spaced data spanning Y (a natural number) years as regularly spaced data consisting of (Y×365)⁄7 (rounded up to the nearest integer) values, that is we treated all data as weekly data. We did not perform preprocessing, including interpolation, on missing values in the regularly spaced data. No normalization, outlier removal, or dimensionality reduction was performed.

**Figure 2.**
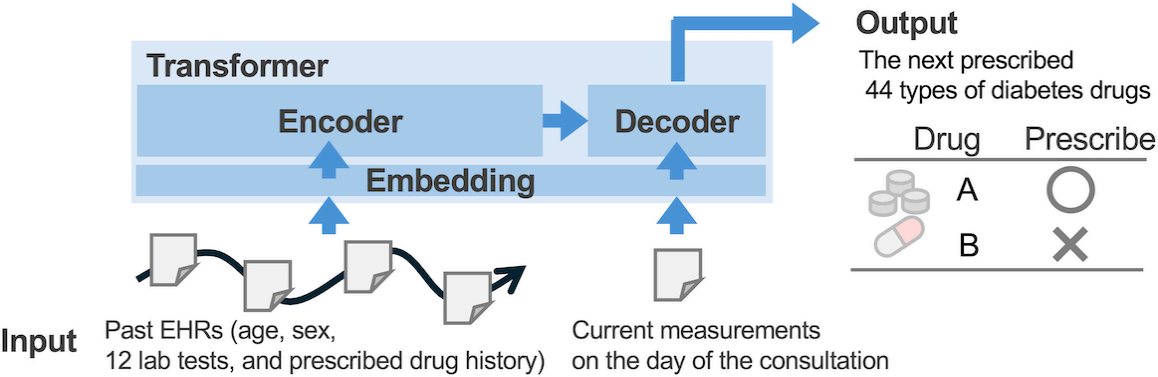
Architecture of the transformer-based encoder-decoder model for predicting the next-prescribed diabetes medications. EHRs: electronic health records.

We designed a transformer-based encoder-decoder model (Figure 2) that takes as input a time series of drugs prescribed and laboratory tests over the past 1 year, sex, and age. The model approaches drug selection as a multichoice task. With N types of drugs, there are 2^N potential prescription combinations. By setting the number of units in the output layer of the transformer decoder to N, we implemented N binary classifications. The model outputs a set of scores representing the probability of each drug being prescribed on that day and, for each drug and day, a binary prescription decision based on whether the prescription probability is greater than or equal to 0.5.

We treated age and HbA_1c_ values as numerical data, sex as a categorical label, and prescription history as a set of categorical labels. In addition, a time series representing the presence or absence of missing data, called an attention mask, was simultaneously generated. Each data type was transformed into a uniform-dimensional vector using an embedding layer specific to that data type, which was then handled by the transformer model. Time information was converted to a uniform-dimensional vector using a positional embedding layer with a periodic function and added to the output value of the embedding layers.

The model incorporates two types of attention layers: self-attention in the encoder, designed to extract relationships in time and meaning from the time series of drugs prescribed and laboratory tests, and cross-attention in the decoder, used to predict the next prescribed drugs based on these relationships. Times in the time series that contain missing data are ignored in the self-attention and mutual attention calculation process using the attention mask. This makes it easy to handle without additional processing for missing data completion. The self-attention weights were optimized through self-supervised learning. This involves the task of predicting both the next laboratory test values and drugs prescribed using a time series of past laboratory tests and drugs prescribed. The cross-attention weights were optimized through supervised learning involving the task of predicting a set of scores representing the probability of each drug being prescribed on that day.

The model consisted of four transformer-encoder layers including four multihead self-attention blocks and four transformer-decoder layers including four multihead cross-attention blocks, along with a hidden layer of dimension 256. Compared to the text data handled by the language model, the prescription drug selection data has a smaller vocabulary (number of drug types) and a shorter time series, so these hyperparameters were set to about one-third the size of the BERT model [34]. The parameters were optimized by Adam with a learning rate of 1e-4, a batch size of 256, and 100 epochs. The loss functions for numerical and categorical data were mean squared error and focal loss [48], respectively. All implementations were written in Python 3.11 (Python Software Foundation) and PyTorch 2.2 (Meta AI).

The model was trained using prescription records from endocrinologists at the University of Tokyo Hospital. As a result, the model generates outputs aligned with the treatment approaches of these specialists.

### Statistical Methods

We analyzed the characteristics of patients in the dataset using means, SD, and frequency counts. We calculated 95% CI using the bootstrap method where applicable. We performed all statistical analyses using custom Python code.

We compared our model with an established ML method recognized for high accuracy. There were validations on similar T2D prediction tasks favored LightGBM [49,50], making it our chosen reference for comparisons. We compared the predictive accuracy of the two methods with the three different training dataset for the prescription prediction of 44 drugs using both macro- and microaverages [51] of the receiver operating characteristic area under the curve (ROC-AUC) as metrics. The macroaverage is a measure of the average performance across all classes independently. The method computes the metrics of ROC-AUC for each individual drug, then averages them to obtain an overall score. This analysis method gives equal weight to each drug, regardless of its size or imbalance in the dataset. On the other hand, the microaverage is a measure of the aggregate performance that weights all instances in the dataset equally. This analysis method first aggregates the true positives, false positives, true negatives, and false negatives across all drugs, and then computes each metric using these aggregated values. The microaverage treats every prediction equally, without considering the kinds of drugs.

The predictive performance of each drug was also evaluated using ROC-AUC.

### Ethical Considerations

This study was approved by the institutional review board of the University of Tokyo School of Medicine (approval number: 10705-(4)) and was conducted in accordance with the Declaration of Helsinki. This was a retrospective, noninterventional database study without patient involvement. Confidentiality was safeguarded by the University of Tokyo Hospital. According to the Guidelines for Epidemiological Studies of the Ministry of Health, Labour and Welfare of Japan, written informed consent was not required. Information about the current study was available to patients on a website, and patients have the right to cease registration of their data at any time [52].

## Results

### Prediction Performance for Various Sizes of Training Data

We assessed different sizes of training data (Table 2). The best prediction performance was obtained from training with the 5 years of data from 2017 to 2021. This version achieved a microaverage (95% CI) ROC-AUC of 0.993 (0.992-0.994) and a macroaverage (95% CI) ROC-AUC of 0.988 (0.980-0.993), and we selected it as our model. Performance was similar when trained using the full range of 10 years of data, from 2012 to 2021, producing somewhat worse results. For all sizes of training data, we met the study’s objectives of achieving an ROC-AUC above 0.95 from our macro- and microaverage evaluations. The prediction accuracy of LightGBM with the 5 years of data had a microaverage ROC AUC of 0.988 (0.985-0.990), and the transformer outperformed LightGBM.

**Table 2.** Overall prediction performance of models for the next-prescribed diabetes drugs, stratified by the training data period. This table compares the overall accuracy of the developed transformer model against a LightGBM model.

	Transformer		LightGBM	
	Microaverage (95% CI)	Macroaverage (95% CI)	Microaverage (95% CI)	Macroaverage (95% CI)
2 years of training data	0.991 (0.990-0.992)	0.981 (0.975-0.988)	0.987 (0.984-0.989)	0.970 (0.941-0.992)
5 years of training data	0.993 (0.992-0.994)	0.988 (0.980-0.993)	0.988 (0.985-0.990)	0.962 (0.916-0.993)
10 years of training data	0.992 (0.990-0.993)	0.987 (0.976-0.994)	0.984 (0.981-0.986)	0.959 (0.921-0.987)

### Prediction Performance for Each Drug

We examined the prediction performance for each of the 44 drugs (Table 3). The drugs in the table are the top 5 and bottom 5 in terms of the number of prescriptions in the 2 years of training data. All results are provided in the supplemental file (Multimedia Appendix 2). We achieved an ROC-AUC above our target of 0.95 for 43 of the 44 drugs when trained with the 5 years of data.

**Table 3.** Prediction performance for drugs when trained with various sizes of training data. The table shows results for the top 5 and bottom 5 most frequently prescribed drugs, showing a comparison of performance between specific drugs and the impact of the data period of the training data on the prediction accuracy of individual drugs.

	Number of prescriptions in 1 year of test data, n (%)	Number of prescriptions in 2 years of training data		Number of prescriptions in 5 years of training data		Number of prescriptions in 10 years of training data	
		ROC-AUC (95% CI)	Accuracy (95% CI)	ROC-AUC (95% CI)	Accuracy (95% CI)	ROC-AUC (95% CI)	Accuracy (95% CI)
Prescribed drug (top 5)							
Metformin hydrochloride, n (%)	1361 (45.55)	0.992 (0.989-0.995)	0.977 (0.971-0.982)	0.992 (0.989-0.995)	0.982 (0.978-0.987)	0.993 (0.990-0.996)	0.980 (0.975-0.985)
Sitagliptin phosphate hydrate, n (%)	425 (14.22)	0.991 (0.985-0.996)	0.988 (0.985-0.992)	0.994 (0.990-0.997)	0.990 (0.987-0.994)	0.996 (0.994-0.998)	0.992 (0.989-0.995)
Insulin aspart (genetical recombination), n (%)	403 (13.49)	0.993 (0.989-0.997)	0.985 (0.981-0.989)	0.996 (0.993-0.998)	0.985 (0.981-0.989)	0.995 (0.991-0.999)	0.993 (0.990-0.996)
Glimepiride, n (%)	358 (11.98)	0.988 (0.979-0.995)	0.991 (0.988-0.994)	0.991 (0.984-0.996)	0.994 (0.991-0.996)	0.995 (0.990-0.999)	0.992 (0.989-0.995)
Pioglitazone hydrochloride, n (%)	244 (8.17)	0.989 (0.978-0.998)	0.993 (0.990-0.996)	0.990 (0.979-0.999)	0.993 (0.990-0.996)	0.995 (0.992-0.997)	0.985 (0.981-0.989)
Prescribed drug (buttom 5)							
Saxagliptin hydrate, n (%)	30 (1.00)	0.978 (0.944-1.000)	0.998 (0.996-0.999)	0.999 (0.999-1.000)	0.999 (0.998-1.000)	0.999 (0.999-1.000)	0.999 (0.998-1.000)
Anagliptin, n (%)	5 (0.17)	0.999 (0.998-1.000)	0.998 (0.996-0.999)	0.999 (0.999-1.000)	0.999 (0.998-1.000)	0.999 (0.998-1.000)	1.000 (0.999-1.000)
Insulin lispro (genetical recombination) [Insulin lispro Biosimilar 1], n (%)	58 (1.94)	0.945 (0.910-0.972)	0.986 (0.981-0.990)	0.848 (0.784-0.904)	0.981 (0.976-0.986)	0.799 (0.735-0.860)	0.981 (0.975-0.986)
Insulin glargine (genetical recombination) [Insulin glargin biosimilar 2], n (%)	1 (0.03)	0.938 (0.500-0.945)	0.996 (0.994-0.998)	0.990 (0.500-0.993)	0.999 (0.998-1.000)	0.938 (0.500-0.945)	1.000 (0.999-1.000)
Glibenclamide, n (%)	11 (0.37)	0.999 (0.999-1.000)	0.999 (0.997-1.000)	0.999 (0.999-1.000)	0.999 (0.997-1.000)	0.973 (0.935-0.999)	0.996 (0.994-0.998)

The only drug that did not achieve the target value was “Insulin lispro (genetical recombination) [Insulin lispro Biosimilar 1].” The prescription of this drug began in 2020. Therefore, the same instances of prescription were present in all three training data periods. For this drug, the model trained on just 2 years of data had the highest ROC-AUC.

### Interpretability

**Figure 3.**
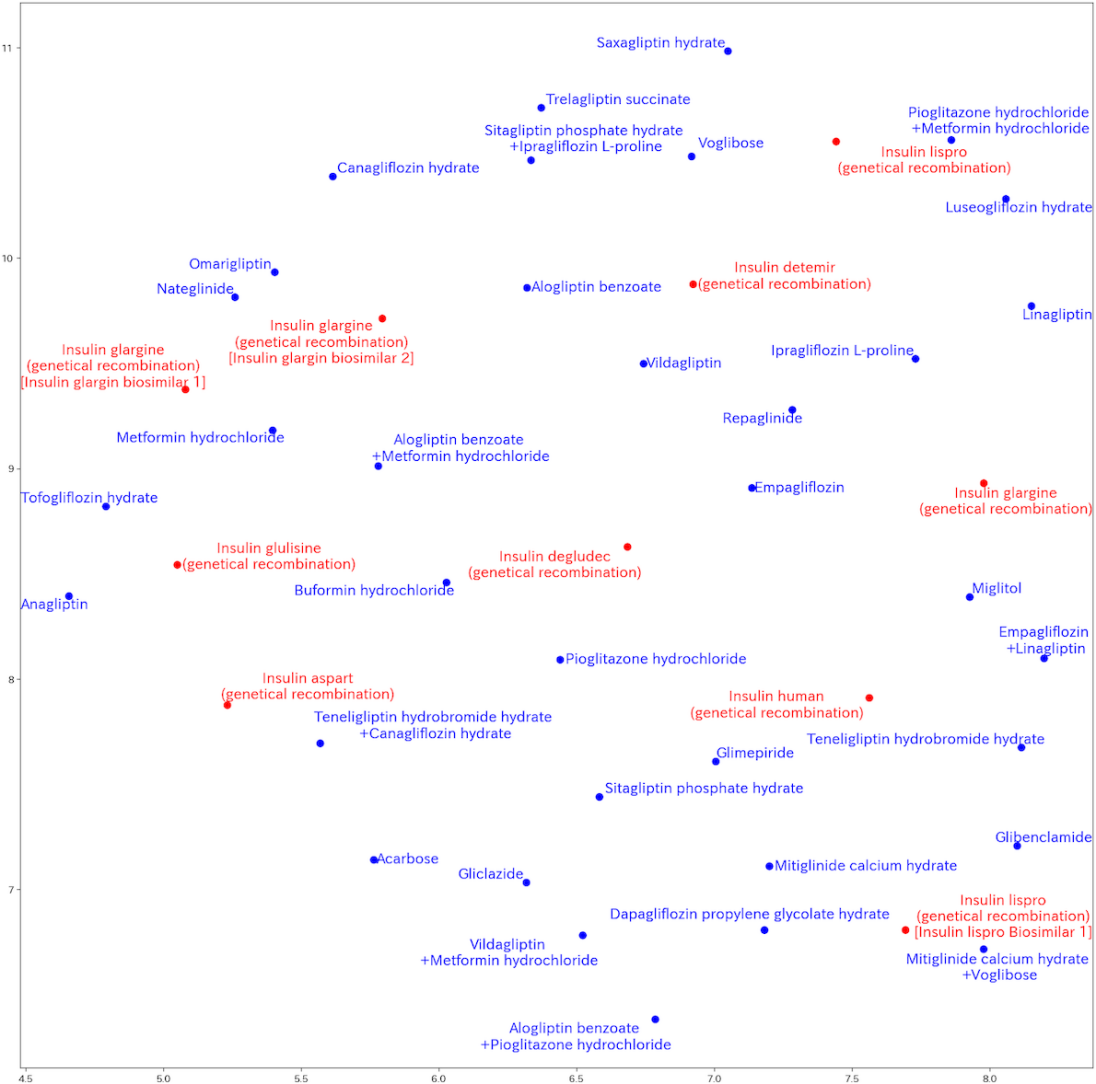
Visualization of learned drug embedding vectors using uniform manifold approximation and projection. This plot displays a 2D representation of the relationships between 44 different diabetes drugs, as learned by the transformer model’s encoder component. Each point corresponds to a specific diabetes drug. Proximity between points suggests that the model identified similarities in how these drugs were used or in the patient contexts associated with their prescription within the training dataset.

The proposed model performed as well as other transformer-based models considering the ability for extracting relationships in time and meaning [53]. The embedding vectors obtained through training represent the closeness of relationships between vectors as proximity. The embedding vectors in this experiment were the same 256 dimensions as the transformer hidden size. Projecting onto two dimensions using uniform manifold approximation and projection [54] allows visualization (Figure 3). While we did not observe a strong tendency for clustering, several biosimilar drugs, such as insulin glargine (genetical recombination, ie, insulin glargin biosimilar 1), were positioned close to the biosimilar drug.

## Discussion

### Evaluation of the Predictive Accuracy

The proposed model achieved impressive predictive accuracy, with macroaverages (95% CI) of ROC-AUC of 0.988 (0.980-0.993). In previous studies [11,12], the ROC-AUC for drugs was less than 0.95, and our model represents a significant improvement relative to this previous work.

The prediction accuracy was higher when trained with short-term data from 5 years than when trained using data from the past 10 years. We suspect that changes in the treatment environment, such as the introduction of new prescription drugs, are responsible for this difference in prediction accuracy. When training on data from the past N years, the model uniformly learns the prescription selection trends for the past N years. Therefore, when N is large, the model is heavily influenced by the trends of older prescription selections. This suspicion is supported by our results that the prescription drug that did not reach target accuracy, “Insulin lispro (genetical recombination) [Insulin lispro Biosimilar 1],” was only recently approved and prescribed. Introduced in 2020, this drug has remained relatively rare in the dataset, as demonstrated in Table 1. Even when expanding the training data period from 2 to 10 years, the number of prescriptions for this drug has remained stagnant at 59, and its proportion within the overall dataset has decreased from 0.23% to a mere 0.03%. This low frequency and temporal bias in the data have rendered learning this specific drug’s patterns challenging. Although ML models generally perform better with more data, for this application it may be better to gather data from more hospitals rather than a longer time span, since it is desirable to learn more fresh data, including new prescription drugs. Transformer models, known for power-law characteristics, benefit from scale-ups [38], and expanding the study to multiple hospitals could explore potential performance enhancements and test the applicability of the power-law in the medical field.

Physicians select drugs considering insulin secretion and insulin resistance, age, obesity, severity of chronic complications, and liver and kidney function. In this experiment, we used age, sex, 12 kinds of diabetes-related laboratory tests, and past prescription history. By adding comprehensive items likely used in medication selection to the model input, performance may be improved.

Our ultimate goal is to improve the treatment outcomes of diabetes. Merely predicting drug selection alone cannot achieve this goal. Expanding the scope to predict the impact of prescribed drugs could further enhance the model’s utility in diabetes treatment.

### Limitations

Our study has notable limitations. First, the model uses only age, sex, 12 kinds of diabetes-related laboratory tests, and past prescription history as inputs. It is desirable to fully account for the various constraints and contraindications that physicians consider in real-world clinical practice. For example, when considering patient characteristics, factors such as age (older adults or pediatric), pregnancy, lactation, BMI, type of diabetes, c-peptide, renal and hepatic function, comorbidities, allergies, urinary tract infection history, diabetic ketoacidosis history, cardiac history, and hypoglycemia must be considered. Regarding medications, considerations include adverse effects, drug resistance, and cost. However, despite these limitations, this study demonstrates that it is possible to narrow down treatment options to some extent using a limited set of variables. We believe that this result holds promise for providing primary care physicians with some guidance and direction. Regarding warnings of constraints and contraindications in drug selection, CDSS have traditionally excelled in this [20,21] and can effectively complement this model. In addition, recent advancements in natural language processing within ML models [33,34] have enabled the extraction of important information on constraints and contraindications from fresh sources such as research papers published daily. We believe there is substantial potential for future research to integrate these important factors into ML models, and we intend to work on this improvement.

Second, the data were sourced from a single hospital, limiting generalizability. Potential biases in the results include the race and geography of the patients and the prescribing tendencies of a limited number of endocrinologists at the hospital. ML models tend to learn biases present in the training data, resulting in predictions that reflect those biases. Prediction accuracy is significantly degraded when different biases exist between the training data and the test data. Therefore, in order to generalize the prediction results of the model, a large dataset of data from multiple hospitals containing data on patients with diverse backgrounds is required. However, in reality, it is difficult to build an ideal dataset due to constraints such as privacy protection and data collection costs. As one solution, we believe that it is good to collect training data that resembles the patterns of patients treated by the primary care physicians who are the users of the model, thereby eliminating biase differences between the training data and the test data as much as possible. Specifically, we collect EHRs of patients who have been involved in prescriptions by endocrinologists at multiple hospitals in a specific region, and use this to build the model that selects prescription drugs for patients treated by primary care physicians in the same region. We believe that this approach will reduce the bias differences in regional characteristics between the training data and the test data, and improve generalization performance with realistic data collection. We would like to verify the validity of this solution in the future.

Third, ML reflects majority characteristics, potentially limiting applicability to diverse patient populations. In the dataset used in the experiment, over 40% (297/637) of patients were prescribed metformin hydrochloride, and patient characteristics are biased. There is a risk that truly effective treatments may not be prescribed correctly to a minority of patients. Prediction failure analysis needs to be further scrutinized, including versus patient characteristics. We should examine this issue by comparing prediction accuracy for each patient cluster.

Fourth, this was a backward-looking study, using past data, and the essential next phase is to assess the model’s predictive capabilities in clinical practice. There is a need for a careful exploration of the model’s effectiveness in real clinical scenarios.

Fifth, while the proposed model requires hyperparameters, we determined these values based on previous studies. Although fine-tuning hyperparameters is generally desirable, transformer models are typically computationally expensive to train, and many previous studies have likewise not fully tuned their parameters. There is potential for performance improvement through hyperparameter tuning, and we intend to investigate this further in future work.

Sixth, the model has poor interpretability. We investigated the proximity relationship between embedding vectors, but no strong tendency was found. It would be better to use other information obtained from the transformer, such as attention weights, to further improve interpretability.

These limitations raise important ethical considerations in the development and application of medical artificial intelligence. Physicians who use the model must be aware of these limitations and exercise appropriate clinical judgment. Obtaining appropriate informed consent from patients is important.

### Conclusions

The proposed model addresses the challenge of predicting the next prescribed drugs. This model, trained using past prescriptions of endocrinologists, has the potential to improve treatment outcomes for nonspecialists by assisting them in making prescription decisions. Future efforts should focus on improving accuracy by incorporating disease state information beyond current inputs and validating the model on large clinical datasets across multiple hospitals.

## Supplementary material

10.2196/67748 Multimedia Appendix 1 Characteristics of patients with diabetes included in the training and testing datasets. Characteristics are presented separately for the patient groups belonging to the different training datasets defined by period: 2 years (2020-2021), 5 years (2017-2021), 10 years (2012-2021) and the independent test dataset (data exclusively from 2022). Variables shown include patient counts, age, sex distribution, laboratory values, and prescription frequencies of 44 drugs.

10.2196/67748 Multimedia Appendix 2 Prediction performance for drugs when trained with various sizes of training data. The table shows results for the 44 prescribed drugs, showing a comparison of performance between specific drugs and the impact of the data period of the training data on the prediction accuracy of individual drugs.

## Abbreviations

CDSS: clinical decision support system
EHR: electronic health record
LightGBM: light gradient boosting machine
ML: machine learning
RNN: recurrent neural network
ROC-AUC: area under the receiver operating characteristic curve
T2D: type 2 diabete
