# Supplementary material for: Enhancing Antidiabetic Drug Selection Using Transformers: Machine-Learning Model Development
Source: JMIR Med Inform. 2025 Jun 2;13:e67748. doi: 10.2196/67748 (PMC12148250; doi:10.2196/67748)
Supplement: Multimedia Appendix 1 [file medinform-v13-e67748-s001.docx]

Multimedia Appendix 1: Characteristics of diabetes patients included in the training and testing datasets. Characteristics are presented separately for the patient groups belonging to the different training datasets defined by period: 2 years (2020-2021), 5 years (2017-2021), 10 years (2012-2021) and the independent test dataset (data exclusively from 2022). Variables shown include patient counts, age, sex distribution, laboratory values, and prescription frequencies of 44 drugs.

|  |  |  | **2-years of training data** | | **5-years of training data** | | **10-years of training data** | | **1-years of test data** | |
| --- | --- | --- | --- | --- | --- | --- | --- | --- | --- | --- |
|  |  |  | Records (N=25484) | Patient (N=3013) | Records (N=78020) | Patient (N=4009) | Records (N=168595) | Patient (N=4524) | Records (N=2988) | Patient (N=637) |
|  |  |  |  |  |  |  |  |  |  |  |
| **Gender** | | |  |  |  |  |  |  |  |  |
|  | Male, n (%) | | 16224 (63.66) | 1915 (63.56) | 49348 (63.25) | 2543 (63.43) | 106782 (63.34) | 2869 (63.42) | 1793 (60.01) | 381 (59.81) |
|  | Female, n (%) | | 9260 (36.34) | 1098 (36.44) | 28672 (36.75) | 1466 (36.57) | 61813 (36.66) | 1655 (36.58) | 1195 (39.99) | 256 (40.19) |
| **Age, mean (SD)** | | | 67.62 (12.59) | 69.06 (12.50) | 67.25 (12.56) | 69.17 (12.87) | 66.41 (12.30) | 68.98 (13.10) | 68.80 (12.31) | 69.68 (12.26) |
| **HbA1c, mean (SD)** | | | 7.37 (1.08) |  | 7.31 (1.06) |  | 7.25 (1.03) |  | 7.31 (1.02) |  |
|  | < 6, n (%) | | 920 (3.61) | 323 (10.72) | 3046 (3.90) | 754 (18.81) | 7286 (4.32) | 1345 (29.73) | 96 (3.21) | 49 (7.69) |
|  | 6-7, n (%) | | 8874 (34.82) | 1865 (61.90) | 29030 (37.21) | 3027 (75.51) | 66470 (39.43) | 3835 (84.77) | 1113 (37.25) | 370 (58.08) |
|  | 7-8, n (%) | | 10177 (39.93) | 2135 (70.86) | 30493 (39.08) | 3157 (78.75) | 64355 (38.17) | 3830 (84.66) | 1189 (39.79) | 404 (63.42) |
|  | >= 8, n (%) | | 5513 (21.63) | 1224 (40.62) | 15451 (19.80) | 2008 (50.09) | 30484 (18.08) | 2725 (60.23) | 590 (19.75) | 200 (31.40) |
|  | missing, n (%) | | 0 (0.00) | 0 (0.00) | 0 (0.00) | 0 (0.00) | 0 (0.00) | 0 (0.00) | 0 (0.00) | 0 (0.00) |
| **T-Cho, mean (SD)** | | | 186.25 (36.26) |  | 185.58 (36.25) |  | 184.30 (35.15) |  | 184.13 (34.12) |  |
|  | < 120, n (%) | | 371 (1.46) | 149 (4.95) | 1091 (1.40) | 318 (7.93) | 2256 (1.34) | 497 (10.99) | 46 (1.54) | 28 (4.40) |
|  | 120-220, n (%) | | 15832 (62.13) | 2291 (76.04) | 49175 (63.03) | 3295 (82.19) | 106123 (62.95) | 3946 (87.22) | 1917 (64.16) | 459 (72.06) |
|  | >= 220, n (%) | | 3233 (12.69) | 838 (27.81) | 9458 (12.12) | 1474 (36.77) | 18367 (10.89) | 2084 (46.07) | 294 (9.84) | 123 (19.31) |
|  | missing, n (%) | | 6048 (23.73) | 572 (18.98) | 18296 (23.45) | 570 (14.22) | 41849 (24.82) | 439 (9.70) | 731 (24.46) | 143 (22.45) |
| **HDL-C, mean (SD)** | | | 60.99 (18.23) |  | 60.07 (17.94) |  | 59.81 (17.80) |  | 63.76 (19.79) |  |
|  | < 40, n (%) | | 1943 (7.62) | 512 (16.99) | 6633 (8.50) | 964 (24.05) | 14694 (8.72) | 1374 (30.37) | 158 (5.29) | 67 (10.52) |
|  | 40-120, n (%) | | 21304 (83.60) | 2737 (90.84) | 63249 (81.07) | 3644 (90.90) | 134924 (80.03) | 4195 (92.73) | 2564 (85.81) | 577 (90.58) |
|  | >= 120, n (%) | | 195 (0.77) | 59 (1.96) | 494 (0.63) | 100 (2.49) | 966 (0.57) | 137 (3.03) | 44 (1.47) | 11 (1.73) |
|  | missing, n (%) | | 2042 (8.01) | 178 (5.91) | 7644 (9.80) | 243 (6.06) | 18011 (10.68) | 219 (4.84) | 222 (7.43) | 40 (6.28) |
| **Cre, mean (SD)** | | | 0.97 (0.69) |  | 0.96 (0.75) |  | 0.95 (0.72) |  | 1.04 (1.08) |  |
|  | Male, mean (SD) | | 1.08 (0.71) |  | 1.07 (0.74) |  | 1.05 (0.74) |  | 1.18 (1.23) |  |
|  |  | < 0.65, n (%) | 758 (2.97) | 191 (6.34) | 2675 (3.43) | 405 (10.10) | 5783 (3.43) | 566 (12.51) | 111 (3.71) | 37 (5.81) |
|  |  | 0.65-1.09, n (%) | 10672 (41.88) | 1482 (49.19) | 32766 (42.00) | 2057 (51.31) | 71870 (42.63) | 2456 (54.29) | 1145 (38.32) | 290 (45.53) |
|  |  | >= 1.09, n (%) | 4543 (17.83) | 750 (24.89) | 13140 (16.84) | 1093 (27.26) | 27001 (16.02) | 1337 (29.55) | 516 (17.27) | 129 (20.25) |
|  |  | missing, n (%) | 251 (0.98) | 6 (0.20) | 767 (0.98) | 5 (0.12) | 2128 (1.26) | 7 (0.15) | 21 (0.70) | 1 (0.16) |
|  | Female, mean (SD) | | 0.78 (0.60) |  | 0.78 (0.72) |  | 0.77 (0.65) |  | 0.82 (0.74) |  |
|  |  | < 0.46, n (%) | 357 (1.40) | 89 (2.95) | 1160 (1.49) | 175 (4.37) | 2616 (1.55) | 282 (6.23) | 28 (0.94) | 11 (1.73) |
|  |  | 0.46-0.82, n (%) | 6596 (25.88) | 895 (29.70) | 20561 (26.35) | 1224 (30.53) | 44130 (26.18) | 1428 (31.56) | 825 (27.61) | 194 (30.46) |
|  |  | >= 0.82, n (%) | 2133 (8.37) | 372 (12.35) | 6370 (8.16) | 596 (14.87) | 13572 (8.05) | 730 (16.14) | 326 (10.91) | 93 (14.60) |
|  |  | missing, n (%) | 174 (0.68) | 11 (0.37) | 581 (0.74) | 11 (0.27) | 1495 (0.89) | 16 (0.35) | 16 (0.54) | 2 (0.31) |
| **Glu, mean (SD)** | | | 147.68 (51.19) |  | 147.00 (51.31) |  | 144.12 (50.77) |  | 145.47 (48.67) |  |
|  | < 70, n (%) | | 302 (1.19) | 169 (5.61) | 979 (1.25) | 403 (10.05) | 2709 (1.61) | 743 (16.42) | 28 (0.94) | 22 (3.45) |
|  | 70-110, n (%) | | 4230 (16.60) | 1389 (46.10) | 13604 (17.44) | 2483 (61.94) | 33633 (19.95) | 3403 (75.22) | 555 (18.57) | 260 (40.82) |
|  | >= 110, n (%) | | 20863 (81.87) | 2924 (97.05) | 63201 (81.01) | 3907 (97.46) | 131586 (78.05) | 4418 (97.66) | 2394 (80.12) | 609 (95.60) |
|  | missing, n (%) | | 89 (0.35) | 7 (0.23) | 236 (0.30) | 5 (0.12) | 667 (0.40) | 6 (0.13) | 11 (0.37) | 2 (0.31) |
| **ALT(GPT), mean (SD)** | | | 25.89 (20.70) |  | 24.93 (20.21) |  | 24.85 (20.02) |  | 23.44 (16.03) |  |
|  | < 4, n (%) | | 20 (0.08) | 8 (0.27) | 20 (0.03) | 8 (0.20) | 39 (0.02) | 11 (0.24) | 1 (0.03) | 1 (0.16) |
|  | 4-44, n (%) | | 21914 (85.99) | 2860 (94.92) | 67558 (86.59) | 3875 (96.66) | 143312 (85.00) | 4408 (97.44) | 2660 (89.02) | 603 (94.66) |
|  | >= 44, n (%) | | 2857 (11.21) | 717 (23.80) | 7924 (10.16) | 1281 (31.95) | 16801 (9.97) | 1935 (42.77) | 265 (8.87) | 109 (17.11) |
|  | missing, n (%) | | 693 (2.72) | 41 (1.36) | 2518 (3.23) | 45 (1.12) | 8443 (5.01) | 34 (0.75) | 62 (2.07) | 4 (0.63) |
| **TG, mean (SD)** | | | 148.77 (118.94) |  | 148.26 (130.87) |  | 145.10 (123.91) |  | 139.82 (105.84) |  |
|  | < 30, n (%) | | 32 (0.13) | 19 (0.63) | 109 (0.14) | 38 (0.95) | 203 (0.12) | 64 (1.41) | 4 (0.13) | 4 (0.63) |
|  | 30-150, n (%) | | 15642 (61.38) | 2536 (84.17) | 48040 (61.57) | 3531 (88.08) | 105601 (62.64) | 4108 (90.80) | 1950 (65.26) | 531 (83.36) |
|  | >= 150, n (%) | | 8685 (34.08) | 1761 (58.45) | 25655 (32.88) | 2650 (66.10) | 53349 (31.64) | 3295 (72.83) | 957 (32.03) | 336 (52.75) |
|  | missing, n (%) | | 1125 (4.41) | 91 (3.02) | 4216 (5.40) | 141 (3.52) | 9442 (5.60) | 146 (3.23) | 77 (2.58) | 13 (2.04) |
| **U-Alb/Cre, mean (SD)** | | | 116.11 (274.55) |  | 117.85 (198.79) |  | 117.78 (175.95) |  | 107.59 (279.90) |  |
|  | < 30, n (%) | | 3311 (12.99) | 905 (30.04) | 9530 (12.21) | 1797 (44.82) | 17821 (10.57) | 2476 (54.73) | 487 (16.30) | 190 (29.83) |
|  | >= 30, n (%) | | 7528 (29.54) | 1828 (60.67) | 21674 (27.78) | 2823 (70.42) | 40215 (23.85) | 3425 (75.71) | 893 (29.89) | 332 (52.12) |
|  | missing, n (%) | | 14645 (57.47) | 780 (25.89) | 46816 (60.01) | 585 (14.59) | 110559 (65.58) | 491 (10.85) | 1608 (53.82) | 192 (30.14) |
| **ALT(GPT), mean (SD)** | | | 25.61 (16.35) |  | 25.31 (15.73) |  | 25.27 (16.69) |  | 23.89 (15.94) |  |
|  | < 7, n (%) | | 8 (0.03) | 6 (0.20) | 23 (0.03) | 11 (0.27) | 31 (0.02) | 15 (0.33) | 4 (0.13) | 2 (0.31) |
|  | 7-38, n (%) | | 21377 (83.88) | 2814 (93.40) | 64976 (83.28) | 3842 (95.83) | 138118 (81.92) | 4382 (96.86) | 2597 (86.91) | 589 (92.46) |
|  | >= 38, n (%) | | 2699 (10.59) | 741 (24.59) | 7870 (10.09) | 1354 (33.77) | 16277 (9.65) | 1996 (44.12) | 241 (8.07) | 100 (15.70) |
|  | missing, n (%) | | 1400 (5.49) | 101 (3.35) | 5151 (6.60) | 88 (2.20) | 14169 (8.40) | 71 (1.57) | 146 (4.89) | 20 (3.14) |
| **γ-GTP, mean (SD)** | | | 42.25 (72.74) |  | 42.16 (71.83) |  | 42.10 (65.12) |  | 43.55 (110.44) |  |
|  | Male, mean (SD) | | 46.61 (83.73) |  | 47.15 (83.46) |  | 47.43 (75.57) |  | 51.49 (138.93) |  |
|  |  | < 80, n (%) | 12863 (50.47) | 1702 (56.49) | 38545 (49.40) | 2305 (57.50) | 80965 (48.02) | 2687 (59.39) | 1468 (49.13) | 332 (52.12) |
|  |  | >= 80, n (%) | 1724 (6.77) | 356 (11.82) | 5303 (6.80) | 689 (17.19) | 11196 (6.64) | 978 (21.62) | 163 (5.46) | 59 (9.26) |
|  |  | missing, n (%) | 1637 (6.42) | 114 (3.78) | 5500 (7.05) | 113 (2.82) | 14621 (8.67) | 68 (1.50) | 162 (5.42) | 21 (3.30) |
|  | Female, mean (SD) | | 34.45 (46.10) |  | 33.50 (43.66) |  | 32.88 (39.55) |  | 31.12 (29.00) |  |
|  |  | < 30, n (%) | 5538 (21.73) | 780 (25.89) | 17127 (21.95) | 1114 (27.79) | 36198 (21.47) | 1311 (28.98) | 741 (24.80) | 180 (28.26) |
|  |  | >= 30, n (%) | 2630 (10.32) | 468 (15.53) | 8141 (10.43) | 771 (19.23) | 17111 (10.15) | 1010 (22.33) | 302 (10.11) | 88 (13.81) |
|  |  | missing, n (%) | 1092 (4.29) | 85 (2.82) | 3404 (4.36) | 71 (1.77) | 8504 (5.04) | 66 (1.46) | 152 (5.09) | 20 (3.14) |
| **protain** | | |  |  |  |  |  |  |  |  |
|  | (-), n (%) | | 15049 (59.05) | 2368 (78.59) | 48424 (62.07) | 3337 (83.24) | 101409 (60.15) | 3947 (87.25) | 1738 (58.17) | 470 (73.78) |
|  | (+-), n (%) | | 2965 (11.63) | 1271 (42.18) | 4770 (6.11) | 1688 (42.11) | 9819 (5.82) | 2330 (51.50) | 427 (14.29) | 225 (35.32) |
|  | (1+), n (%) | | 2435 (9.56) | 904 (30.00) | 5792 (7.42) | 1460 (36.42) | 11269 (6.68) | 2044 (45.18) | 296 (9.91) | 143 (22.45) |
|  | (2+), n (%) | | 1612 (6.33) | 495 (16.43) | 4712 (6.04) | 915 (22.82) | 10092 (5.99) | 1321 (29.20) | 225 (7.53) | 88 (13.81) |
|  | (3+), n (%) | | 691 (2.71) | 231 (7.67) | 2581 (3.31) | 428 (10.68) | 5702 (3.38) | 637 (14.08) | 43 (1.44) | 23 (3.61) |
|  | (4+), n (%) | | 170 (0.67) | 54 (1.79) | 389 (0.50) | 114 (2.84) | 772 (0.46) | 178 (3.93) | 18 (0.60) | 12 (1.88) |
|  | missing, n (%) | | 2562 (10.05) | 187 (6.21) | 11352 (14.55) | 184 (4.59) | 29532 (17.52) | 127 (2.81) | 241 (8.07) | 30 (4.71) |
| **glycogen** | | |  |  |  |  |  |  |  |  |
|  | (-), n (%) | | 11470 (45.01) | 1976 (65.58) | 38479 (49.32) | 3297 (82.24) | 92788 (55.04) | 4163 (92.02) | 1338 (44.78) | 371 (58.24) |
|  | (+-), n (%) | | 731 (2.87) | 486 (16.13) | 731 (0.94) | 486 (12.12) | 997 (0.59) | 678 (14.99) | 104 (3.48) | 86 (13.50) |
|  | (1+), n (%) | | 984 (3.86) | 617 (20.48) | 3630 (4.65) | 1447 (36.09) | 8917 (5.29) | 2280 (50.40) | 92 (3.08) | 69 (10.83) |
|  | (2+), n (%) | | 821 (3.22) | 526 (17.46) | 2684 (3.44) | 1225 (30.56) | 6330 (3.75) | 1916 (42.35) | 87 (2.91) | 68 (10.68) |
|  | (3+), n (%) | | 1830 (7.18) | 922 (30.60) | 3902 (5.00) | 1519 (37.89) | 7152 (4.24) | 2027 (44.81) | 178 (5.96) | 124 (19.47) |
|  | (4+), n (%) | | 7086 (27.81) | 1255 (41.65) | 17242 (22.10) | 1821 (45.42) | 22879 (13.57) | 2237 (49.45) | 948 (31.73) | 265 (41.60) |
|  | missing, n (%) | | 2562 (10.05) | 187 (6.21) | 11352 (14.55) | 184 (4.59) | 29532 (17.52) | 127 (2.81) | 241 (8.07) | 30 (4.71) |
| **Prescribed drug** | | |  |  |  |  |  |  |  |  |
|  | Metformin hydrochloride, n (%) | | 11257 | 11257 (44.17) | 1390 (46.13) | 33442 (42.86) | 1952 (48.69) | 72337 (42.91) | 2376 (52.52) | 1361 (45.55) |
|  | Sitagliptin phosphate hydrate, n (%) | | 4963 | 4963 (19.47) | 693 (23.00) | 15420 (19.76) | 1148 (28.64) | 37438 (22.21) | 1808 (39.96) | 425 (14.22) |
|  | Insulin aspart (genetical recombination), n (%) | | 3212 | 3212 (12.60) | 377 (12.51) | 10039 (12.87) | 603 (15.04) | 20149 (11.95) | 833 (18.41) | 403 (13.49) |
|  | Glimepiride, n (%) | | 2885 | 2885 (11.32) | 398 (13.21) | 10387 (13.31) | 690 (17.21) | 28855 (17.11) | 1141 (25.22) | 358 (11.98) |
|  | Pioglitazone hydrochloride, n (%) | | 2731 | 2731 (10.72) | 354 (11.75) | 9450 (12.11) | 592 (14.77) | 24305 (14.42) | 910 (20.11) | 244 (8.17) |
|  | Empagliflozin, n (%) | | 2586 | 2586 (10.15) | 334 (11.09) | 5504 (7.05) | 447 (11.15) | 5676 (3.37) | 456 (10.08) | 387 (12.95) |
|  | Voglibose, n (%) | | 2403 | 2403 (9.43) | 294 (9.76) | 8210 (10.52) | 514 (12.82) | 19492 (11.56) | 769 (17.00) | 237 (7.93) |
|  | Insulin degludec (genetical recombination), n (%) | | 2073 | 2073 (8.13) | 279 (9.26) | 5904 (7.57) | 413 (10.30) | 8520 (5.05) | 460 (10.17) | 298 (9.97) |
|  | Miglitol, n (%) | | 2037 | 2037 (7.99) | 247 (8.20) | 6792 (8.71) | 395 (9.85) | 15862 (9.41) | 597 (13.20) | 303 (10.14) |
|  | Dapagliflozin propylene glycolate hydrate, n (%) | | 1979 | 1979 (7.77) | 272 (9.03) | 4028 (5.16) | 334 (8.33) | 4417 (2.62) | 346 (7.65) | 239 (8.00) |
|  | Vildagliptin + Metformin hydrochloride, n (%) | | 1922 | 1922 (7.54) | 255 (8.46) | 4700 (6.02) | 323 (8.06) | 4995 (2.96) | 332 (7.34) | 163 (5.46) |
|  | Insulin glargine (genetical recombination) [Insulin glargin biosimilar 1], n (%) | | 1827 | 1827 (7.17) | 273 (9.06) | 5683 (7.28) | 486 (12.12) | 6240 (3.70) | 521 (11.52) | 258 (8.63) |
|  | Linagliptin, n (%) | | 1737 | 1737 (6.82) | 254 (8.43) | 6344 (8.13) | 486 (12.12) | 12234 (7.26) | 698 (15.43) | 258 (8.63) |
|  | Insulin lispro (genetical recombination), n (%) | | 1576 | 1576 (6.18) | 214 (7.10) | 4758 (6.10) | 351 (8.76) | 9519 (5.65) | 468 (10.34) | 221 (7.40) |
|  | Vildagliptin, n (%) | | 1441 | 1441 (5.65) | 222 (7.37) | 5827 (7.47) | 455 (11.35) | 19168 (11.37) | 885 (19.56) | 134 (4.48) |
|  | Insulin glargine (genetical recombination), n (%) | | 1394 | 1394 (5.47) | 186 (6.17) | 4339 (5.56) | 329 (8.21) | 18111 (10.74) | 972 (21.49) | 237 (7.93) |
|  | Repaglinide, n (%) | | 1328 | 1328 (5.21) | 197 (6.54) | 3548 (4.55) | 303 (7.56) | 5081 (3.01) | 367 (8.11) | 160 (5.35) |
|  | Teneligliptin hydrobromide hydrate, n (%) | | 1252 | 1252 (4.91) | 181 (6.01) | 4050 (5.19) | 323 (8.06) | 6530 (3.87) | 417 (9.22) | 143 (4.79) |
|  | Gliclazide, n (%) | | 1200 | 1200 (4.71) | 191 (6.34) | 3674 (4.71) | 320 (7.98) | 9117 (5.41) | 487 (10.76) | 133 (4.45) |
|  | Ipragliflozin L-proline, n (%) | | 1155 | 1155 (4.53) | 163 (5.41) | 3102 (3.98) | 233 (5.81) | 3695 (2.19) | 262 (5.79) | 138 (4.62) |
|  | Alogliptin benzoate, n (%) | | 974 | 974 (3.82) | 147 (4.88) | 3827 (4.91) | 292 (7.28) | 11746 (6.97) | 551 (12.18) | 125 (4.18) |
|  | Canagliflozin hydrate, n (%) | | 929 | 929 (3.65) | 116 (3.85) | 2253 (2.89) | 160 (3.99) | 2468 (1.46) | 166 (3.67) | 74 (2.48) |
|  | Mitiglinide calcium hydrate, n (%) | | 884 | 884 (3.47) | 144 (4.78) | 2690 (3.45) | 269 (6.71) | 5333 (3.16) | 424 (9.37) | 111 (3.71) |
|  | Alogliptin benzoate + Pioglitazone hydrochloride, n (%) | | 626 | 626 (2.46) | 76 (2.52) | 2195 (2.81) | 118 (2.94) | 4674 (2.77) | 167 (3.69) | 70 (2.34) |
|  | Alogliptin benzoate + Metformin hydrochloride, n (%) | | 543 | 543 (2.13) | 76 (2.52) | 1010 (1.29) | 107 (2.67) | 1010 (0.60) | 107 (2.37) | 80 (2.68) |
|  | Mitiglinide calcium hydrate + Voglibose, n (%) | | 522 | 522 (2.05) | 72 (2.39) | 1333 (1.71) | 109 (2.72) | 2209 (1.31) | 141 (3.12) | 69 (2.31) |
|  | Insulin human (genetical recombination), n (%) | | 491 | 491 (1.93) | 66 (2.19) | 1652 (2.12) | 109 (2.72) | 5135 (3.05) | 213 (4.71) | 37 (1.24) |
|  | Sitagliptin phosphate hydrate + Ipragliflozin L-proline, n (%) | | 444 | 444 (1.74) | 62 (2.06) | 661 (0.85) | 74 (1.85) | 661 (0.39) | 74 (1.64) | 41 (1.37) |
|  | Insulin detemir (genetical recombination), n (%) | | 421 | 421 (1.65) | 56 (1.86) | 1385 (1.78) | 105 (2.62) | 4423 (2.62) | 197 (4.35) | 29 (0.97) |
|  | Omarigliptin, n (%) | | 374 | 374 (1.47) | 58 (1.92) | 1442 (1.85) | 105 (2.62) | 1467 (0.87) | 108 (2.39) | 50 (1.67) |
|  | Empagliflozin + Linagliptin, n (%) | | 361 | 361 (1.42) | 56 (1.86) | 441 (0.57) | 60 (1.50) | 441 (0.26) | 60 (1.33) | 44 (1.47) |
|  | Luseogliflozin hydrate, n (%) | | 351 | 351 (1.38) | 46 (1.53) | 1034 (1.33) | 74 (1.85) | 1188 (0.70) | 79 (1.75) | 64 (2.14) |
|  | Tofogliflozin hydrate, n (%) | | 339 | 339 (1.33) | 51 (1.69) | 605 (0.78) | 69 (1.72) | 633 (0.38) | 71 (1.57) | 61 (2.04) |
|  | Insulin glulisine (genetical recombination), n (%) | | 335 | 335 (1.31) | 39 (1.29) | 1159 (1.49) | 66 (1.65) | 2750 (1.63) | 108 (2.39) | 44 (1.47) |
|  | Pioglitazone hydrochloride + Metformin hydrochloride, n (%) | | 289 | 289 (1.13) | 39 (1.29) | 817 (1.05) | 58 (1.45) | 1825 (1.08) | 84 (1.86) | 35 (1.17) |
|  | Acarbose, n (%) | | 279 | 279 (1.09) | 33 (1.10) | 1001 (1.28) | 53 (1.32) | 2864 (1.70) | 89 (1.97) | 17 (0.57) |
|  | Teneligliptin hydrobromide hydrate + Canagliflozin hydrate, n (%) | | 214 | 214 (0.84) | 31 (1.03) | 396 (0.51) | 37 (0.92) | 396 (0.23) | 37 (0.82) | 47 (1.57) |
|  | Trelagliptin succinate, n (%) | | 188 | 188 (0.74) | 29 (0.96) | 762 (0.98) | 62 (1.55) | 828 (0.49) | 66 (1.46) | 33 (1.10) |
|  | Nateglinide, n (%) | | 159 | 159 (0.62) | 25 (0.83) | 730 (0.94) | 55 (1.37) | 2042 (1.21) | 102 (2.25) | 15 (0.50) |
|  | Saxagliptin hydrate, n (%) | | 158 | 158 (0.62) | 17 (0.56) | 586 (0.75) | 41 (1.02) | 960 (0.57) | 52 (1.15) | 30 (1.00) |
|  | Anagliptin, n (%) | | 142 | 142 (0.56) | 17 (0.56) | 528 (0.68) | 34 (0.85) | 986 (0.58) | 51 (1.13) | 5 (0.17) |
|  | Insulin lispro (genetical recombination) [Insulin lispro Biosimilar 1], n (%) | | 59 | 59 (0.23) | 19 (0.63) | 59 (0.08) | 19 (0.47) | 59 (0.03) | 19 (0.42) | 58 (1.94) |
|  | Insulin glargine (genetical recombination) [Insulin glargin biosimilar 2], n (%) | | 39 | 39 (0.15) | 9 (0.30) | 86 (0.11) | 14 (0.35) | 86 (0.05) | 14 (0.31) | 1 (0.03) |
|  | Glibenclamide, n (%) | | 33 | 33 (0.13) | 5 (0.17) | 280 (0.36) | 24 (0.60) | 1722 (1.02) | 87 (1.92) | 11 (0.37) |
