# Supplementary material for: Enhancing Antidiabetic Drug Selection Using Transformers: Machine-Learning Model Development
Source: JMIR Med Inform. 2025 Jun 2;13:e67748. doi: 10.2196/67748 (PMC12148250; doi:10.2196/67748)
Supplement: Multimedia Appendix 2 [file medinform-v13-e67748-s002.docx]

Multimedia Appendix 2: Prediction performance for drugs when trained with various sizes of training data. The table shows results for the 44 prescribed drugs, showing a comparison of performance between specific drugs and the impact of the data period of the training data on the prediction accuracy of individual drugs.

| Drug | No. of prescriptions, n (%) | 2-years of training data | | 5-years of training data | | 10-years of training data | |
| --- | --- | --- | --- | --- | --- | --- | --- |
|  |  | ROC AUC | Accuracy | ROC AUC | Accuracy | ROC AUC | Accuracy |
|  |  |  |  |  |  |  |  |
| Metformin hydrochloride, n (%) | 1361 (45.55) | 0.992 (0.989, 0.995) | 0.977 (0.971, 0.982) | 0.992 (0.989, 0.995) | 0.982 (0.978, 0.987) | 0.993 (0.990, 0.996) | 0.980 (0.975, 0.985) |
| Sitagliptin phosphate hydrate, n (%) | 425 (14.22) | 0.991 (0.985, 0.996) | 0.988 (0.985, 0.992) | 0.994 (0.990, 0.997) | 0.990 (0.987, 0.994) | 0.996 (0.994, 0.998) | 0.992 (0.989, 0.995) |
| Insulin aspart (genetical recombination), n (%) | 403 (13.49) | 0.993 (0.989, 0.997) | 0.985 (0.981, 0.989) | 0.996 (0.993, 0.998) | 0.985 (0.981, 0.989) | 0.995 (0.991, 0.999) | 0.993 (0.990, 0.996) |
| Glimepiride, n (%) | 358 (11.98) | 0.988 (0.979, 0.995) | 0.991 (0.988, 0.994) | 0.991 (0.984, 0.996) | 0.994 (0.991, 0.996) | 0.995 (0.990, 0.999) | 0.992 (0.989, 0.995) |
| Pioglitazone hydrochloride, n (%) | 244 (8.17) | 0.989 (0.978, 0.998) | 0.993 (0.990, 0.996) | 0.990 (0.979, 0.999) | 0.993 (0.990, 0.996) | 0.995 (0.992, 0.997) | 0.985 (0.981, 0.989) |
| Empagliflozin, n (%) | 387 (12.95) | 0.998 (0.994, 1.000) | 0.998 (0.996, 0.999) | 0.991 (0.984, 0.997) | 0.993 (0.990, 0.996) | 0.992 (0.985, 0.997) | 0.990 (0.986, 0.994) |
| Voglibose, n (%) | 237 (7.93) | 0.998 (0.995, 1.000) | 0.994 (0.991, 0.997) | 0.996 (0.991, 0.999) | 0.996 (0.994, 0.998) | 0.997 (0.994, 1.000) | 0.996 (0.993, 0.998) |
| Insulin degludec (genetical recombination), n (%) | 298 (9.97) | 0.995 (0.991, 0.997) | 0.985 (0.980, 0.989) | 0.995 (0.990, 0.998) | 0.991 (0.987, 0.994) | 0.994 (0.981, 1.000) | 0.998 (0.996, 0.999) |
| Miglitol, n (%) | 303 (10.14) | 0.993 (0.985, 0.999) | 0.996 (0.994, 0.998) | 0.999 (0.998, 1.000) | 0.997 (0.995, 0.999) | 0.999 (0.998, 1.000) | 0.997 (0.995, 0.999) |
| Dapagliflozin propylene glycolate hydrate, n (%) | 239 (8.00) | 0.993 (0.984, 0.999) | 0.996 (0.994, 0.998) | 0.979 (0.964, 0.990) | 0.991 (0.988, 0.994) | 0.991 (0.979, 0.999) | 0.997 (0.995, 0.999) |
| Vildagliptin + Metformin hydrochloride, n (%) | 163 (5.46) | 0.988 (0.972, 0.998) | 0.996 (0.994, 0.998) | 0.991 (0.983, 0.997) | 0.992 (0.988, 0.995) | 0.954 (0.897, 0.996) | 0.995 (0.992, 0.997) |
| Insulin glargine (genetical recombination) [Insulin glargin biosimilar 1], n (%) | 258 (8.63) | 0.975 (0.959, 0.989) | 0.989 (0.985, 0.992) | 0.995 (0.991, 0.997) | 0.985 (0.980, 0.989) | 0.993 (0.986, 0.999) | 0.995 (0.992, 0.997) |
| Linagliptin, n (%) | 258 (8.63) | 0.987 (0.978, 0.994) | 0.979 (0.973, 0.984) | 0.994 (0.986, 0.999) | 0.996 (0.994, 0.998) | 0.993 (0.990, 0.995) | 0.977 (0.971, 0.982) |
| Insulin lispro (genetical recombination), n (%) | 221 (7.40) | 0.982 (0.964, 0.995) | 0.995 (0.992, 0.997) | 0.992 (0.983, 0.998) | 0.993 (0.989, 0.996) | 0.989 (0.975, 0.998) | 0.993 (0.989, 0.996) |
| Vildagliptin, n (%) | 134 (4.48) | 0.998 (0.997, 0.999) | 0.994 (0.992, 0.997) | 0.999 (0.997, 0.999) | 0.997 (0.995, 0.999) | 0.999 (0.998, 1.000) | 0.997 (0.995, 0.999) |
| Insulin glargine (genetical recombination), n (%) | 237 (7.93) | 0.991 (0.983, 0.997) | 0.991 (0.987, 0.994) | 0.992 (0.989, 0.994) | 0.972 (0.966, 0.978) | 0.994 (0.986, 0.999) | 0.996 (0.993, 0.998) |
| Repaglinide, n (%) | 160 (5.35) | 0.985 (0.972, 0.996) | 0.993 (0.990, 0.996) | 0.992 (0.981, 0.998) | 0.993 (0.990, 0.996) | 0.990 (0.982, 0.997) | 0.993 (0.990, 0.996) |
| Teneligliptin hydrobromide hydrate, n (%) | 143 (4.79) | 0.989 (0.977, 0.999) | 0.994 (0.991, 0.997) | 0.997 (0.991, 1.000) | 0.997 (0.994, 0.999) | 0.997 (0.992, 1.000) | 0.998 (0.997, 1.000) |
| Gliclazide, n (%) | 133 (4.45) | 0.989 (0.983, 0.994) | 0.977 (0.972, 0.983) | 0.980 (0.963, 0.994) | 0.993 (0.989, 0.996) | 0.995 (0.993, 0.997) | 0.983 (0.979, 0.988) |
| Ipragliflozin L-proline, n (%) | 138 (4.62) | 0.984 (0.966, 0.995) | 0.987 (0.982, 0.991) | 0.994 (0.987, 0.999) | 0.996 (0.993, 0.998) | 0.990 (0.979, 0.997) | 0.993 (0.990, 0.996) |
| Alogliptin benzoate, n (%) | 125 (4.18) | 0.996 (0.989, 1.000) | 0.997 (0.995, 0.999) | 0.997 (0.993, 0.999) | 0.997 (0.994, 0.999) | 0.980 (0.960, 0.996) | 0.991 (0.988, 0.995) |
| Canagliflozin hydrate, n (%) | 74 (2.48) | 0.985 (0.963, 0.999) | 0.997 (0.995, 0.999) | 0.998 (0.996, 0.999) | 0.995 (0.992, 0.997) | 0.998 (0.993, 1.000) | 0.998 (0.997, 1.000) |
| Mitiglinide calcium hydrate, n (%) | 111 (3.71) | 0.998 (0.995, 1.000) | 0.998 (0.996, 0.999) | 0.981 (0.956, 0.997) | 0.991 (0.988, 0.994) | 0.995 (0.991, 0.998) | 0.993 (0.990, 0.996) |
| Alogliptin benzoate + Pioglitazone hydrochloride, n (%) | 70 (2.34) | 0.985 (0.953, 0.999) | 0.994 (0.991, 0.997) | 0.998 (0.996, 1.000) | 0.998 (0.996, 0.999) | 0.970 (0.951, 0.986) | 0.987 (0.983, 0.991) |
| Alogliptin benzoate + Metformin hydrochloride, n (%) | 80 (2.68) | 0.959 (0.883, 0.999) | 0.997 (0.995, 0.998) | 0.996 (0.994, 0.998) | 0.992 (0.989, 0.995) | 1.000 (0.999, 1.000) | 0.999 (0.998, 1.000) |
| Mitiglinide calcium hydrate + Voglibose, n (%) | 69 (2.31) | 0.990 (0.974, 0.998) | 0.994 (0.991, 0.996) | 0.995 (0.988, 0.999) | 0.996 (0.993, 0.998) | 0.996 (0.988, 1.000) | 0.997 (0.995, 0.999) |
| Insulin human (genetical recombination), n (%) | 37 (1.24) | 0.985 (0.962, 0.999) | 0.997 (0.995, 0.999) | 0.990 (0.974, 0.999) | 0.995 (0.992, 0.997) | 0.995 (0.989, 0.998) | 0.994 (0.991, 0.997) |
| Sitagliptin phosphate hydrate + Ipragliflozin L-proline, n (%) | 41 (1.37) | 0.947 (0.919, 0.973) | 0.994 (0.991, 0.997) | 0.999 (0.998, 1.000) | 0.999 (0.997, 1.000) | 0.999 (0.998, 1.000) | 0.998 (0.997, 1.000) |
| Insulin detemir (genetical recombination), n (%) | 29 (0.97) | 0.999 (0.998, 1.000) | 0.997 (0.995, 0.999) | 0.970 (0.918, 0.999) | 0.995 (0.993, 0.998) | 1.000 (1.000, 1.000) | 1.000 (0.999, 1.000) |
| Omarigliptin, n (%) | 50 (1.67) | 0.950 (0.870, 0.998) | 0.996 (0.993, 0.998) | 1.000 (1.000, 1.000) | 1.000 (1.000, 1.000) | 0.994 (0.980, 1.000) | 0.999 (0.998, 1.000) |
| Empagliflozin + Linagliptin, n (%) | 44 (1.47) | 0.915 (0.842, 0.979) | 0.995 (0.992, 0.997) | 0.974 (0.937, 1.000) | 0.998 (0.996, 0.999) | 0.964 (0.914, 0.996) | 0.995 (0.992, 0.997) |
| Luseogliflozin hydrate, n (%) | 64 (2.14) | 0.999 (0.997, 1.000) | 0.997 (0.995, 0.999) | 0.997 (0.993, 0.999) | 0.998 (0.996, 0.999) | 0.998 (0.997, 0.999) | 0.997 (0.995, 0.999) |
| Tofogliflozin hydrate, n (%) | 61 (2.04) | 0.964 (0.880, 1.000) | 0.998 (0.996, 0.999) | 0.972 (0.939, 0.996) | 0.993 (0.990, 0.996) | 0.997 (0.995, 0.999) | 0.997 (0.994, 0.999) |
| Insulin glulisine (genetical recombination), n (%) | 44 (1.47) | 1.000 (1.000, 1.000) | 1.000 (1.000, 1.000) | 0.989 (0.977, 0.998) | 0.995 (0.992, 0.997) | 0.998 (0.996, 0.999) | 0.997 (0.994, 0.999) |
| Pioglitazone hydrochloride + Metformin hydrochloride, n (%) | 35 (1.17) | 0.992 (0.982, 0.998) | 0.997 (0.995, 0.999) | 0.999 (0.999, 1.000) | 0.999 (0.997, 1.000) | 0.998 (0.993, 1.000) | 0.998 (0.996, 0.999) |
| Acarbose, n (%) | 17 (0.57) | 0.984 (0.968, 0.998) | 0.997 (0.995, 0.999) | 0.970 (0.909, 1.000) | 0.998 (0.996, 0.999) | 0.999 (0.999, 1.000) | 0.999 (0.998, 1.000) |
| Teneligliptin hydrobromide hydrate + Canagliflozin hydrate, n (%) | 47 (1.57) | 0.908 (0.772, 1.000) | 0.998 (0.996, 0.999) | 0.998 (0.997, 0.999) | 0.996 (0.994, 0.998) | 0.998 (0.996, 0.999) | 0.997 (0.994, 0.999) |
| Trelagliptin succinate, n (%) | 33 (1.10) | 0.998 (0.996, 0.999) | 0.993 (0.990, 0.996) | 1.000 (0.999, 1.000) | 0.999 (0.998, 1.000) | 0.993 (0.989, 0.997) | 0.992 (0.989, 0.995) |
| Nateglinide, n (%) | 15 (0.50) | 0.969 (0.935, 0.995) | 0.992 (0.989, 0.995) | 0.953 (0.845, 1.000) | 0.997 (0.995, 0.999) | 0.999 (0.998, 0.999) | 0.997 (0.995, 0.999) |
| Saxagliptin hydrate, n (%) | 30 (1.00) | 0.978 (0.944, 1.000) | 0.998 (0.996, 0.999) | 0.999 (0.999, 1.000) | 0.999 (0.998, 1.000) | 0.999 (0.999, 1.000) | 0.999 (0.998, 1.000) |
| Anagliptin, n (%) | 5 (0.17) | 0.999 (0.998, 1.000) | 0.998 (0.996, 0.999) | 0.999 (0.999, 1.000) | 0.999 (0.998, 1.000) | 0.999 (0.998, 1.000) | 1.000 (0.999, 1.000) |
| Insulin lispro (genetical recombination) [Insulin lispro Biosimilar 1], n (%) | 58 (1.94) | 0.945 (0.910, 0.972) | 0.986 (0.981, 0.990) | 0.848 (0.784, 0.904) | 0.981 (0.976, 0.986) | 0.799 (0.735, 0.860) | 0.981 (0.975, 0.986) |
| Insulin glargine (genetical recombination) [Insulin glargin biosimilar 2], n (%) | 1 (0.03) | 0.938 (0.500, 0.945) | 0.996 (0.994, 0.998) | 0.990 (0.500, 0.993) | 0.999 (0.998, 1.000) | 0.938 (0.500, 0.945) | 1.000 (0.999, 1.000) |
| Glibenclamide, n (%) | 11 (0.37) | 0.999 (0.999, 1.000) | 0.999 (0.997, 1.000) | 0.999 (0.999, 1.000) | 0.999 (0.997, 1.000) | 0.973 (0.935, 0.999) | 0.996 (0.994, 0.998) |
